# Clinico-pathologic determinants of non-e-curative outcome following *en*-bloc endoscopic submucosal dissection in patients with early gastric neoplasia

**DOI:** 10.1186/s12885-020-07762-9

**Published:** 2021-01-22

**Authors:** Kidane Siele Embaye, Chao Zhang, Matiwos Araya Ghebrehiwet, Zhihao Wang, Fengdi Zhang, Liwei Liu, Shenghui Qin, Lingzhi Qin, Jun Wang, Xi Wang

**Affiliations:** 1 Institute of Pathology, Tongji Hospital, Tongji Medical College, Huazhong University of Science and Technology, Wuhan, 430030 China; 2 Orotta College of Medicine and Health Sciences, Asmara, Eritrea; 3 Wuhan Third Hospital (Tongren Hospital of Wuhan University), Wuhan, 430060 China

**Keywords:** Early gastric cancer, ESD, Clinico-pathologic factors, Non-e-curative resection

## Abstract

**Background:**

Endoscopic submucosal dissection (ESD) is gaining enormous popularity in the treatment of early gastric cancers (EGCs) in many institutions across the world. However, appropriate selection of candidates for endoscopic resection is crucial to sufficiently mitigate non-e-curative (NEC) resection. This study aims at identifying the various clinico-pathologic factors that independently predict the NEC outcome and depth of submucosal invasion following ESD procedure in patients with EGC.

**Methods:**

Multiple logistic regression analysis was applied to investigate factors that independently predict both non-curability phenomenon and the level of submucosal invasion in patients with early gastric neoplasia. Statistical Packages for the Social Sciences version 23 was used for analysis.

**Results:**

A total of 153 patients (162 EGC lesions) underwent *en*-bloc ESD after which the rate of complete resection and non-e-curative outcome were 95% and 22.2%, correspondingly. Multivariate analysis depicted that tumor location in the upper two third of stomach (odds ratio [OR], 5.46; 95% confidence interval [95% CI], 1.65–18.12; *p* = 0.006), tumor size > 2 cm (OR, 7.63; 95% CI, 2.29–25.42; *p* = 0.001), histologically undifferentiated tumor (OR, 15.54; 95% CI, 1.65–146.22; *p* = 0.001), and tumors with 0-IIa/0-IIc or their mixed variants with predominant 0-IIa/0-IIc (OR, 9.77; 95% CI, 1.23–77.65; *p* = 0.031) were all independent predictors of NEC resection for early gastric tumors. Additionally, location in the upper two third of the stomach (OR, 8.88; 95% CI, 2.90–27.17; *p* < 0.001), ulcerated lesions (OR, 3.70; 95% CI, 1.15–11.90; *p* = 0.028), lesions with > 2 cm (OR, 2.94; 95% CI, 1.08–8.02; *p* = 0.036) and those with poor differentiation (OR, 6.51; 95% CI, 2.23–18.98; *p* = 0.001) were found to have significant association with submucosal invasion.

**Conclusions:**

Tumors located in the upper two third of the stomach having a larger size (> 2 cm), poor histo-differentiation and a gross type of 0-IIa/0-IIc or their mixed variants with predominant 0-IIa/0-IIc were significantly associated with a risk of NEC after ESD procedure. Thus, early gastric tumors displaying these features need to be handled carefully during endoscopic resection. Our findings may shed light on the pre-procedural detection of clinicopathologic factors that determine non-e-curability in patients with EGC.

## Background

Gastric cancer remains to be the fifth most common malignancy and the third leading cause of cancer-related deaths across the globe, and exhibits significant epidemiological variation as a function of age and geography [1]. Cancers of the stomach are two times higher in males than in females and the East Asian countries possess a comparatively higher burden of cases than North American, European and African countries [1, 2]. A subset of these cancers confined to the submucosal layer are called early gastric cancers (EGCs) irrespective of lymph node metastasis [3]. According to Japanese Gastric Cancer Association (JGCA) [3] classification scheme, EGCs belong to class zero (‘0’), which are further subdivided into specific subtypes. These class ‘0’ lesions include protruding type (0-I), superficial lesions with elevation (0-IIa), superficial flat (0-IIb), superficial depressed (0-IIc) and the last type encompasses excavated lesions (0-III). In practice, however, the occurrence of combined macroscopic features is not uncommon, where the more dominant type is written first followed by the other [4].

Despite being a gold standard method in the treatment of early stage gastric cancers for the last many decades, gastrectomy has nowadays been replaced by a relatively less invasive therapeutic endoscopy that has promising clinical outcomes [5, 6]. Endoscopic approach of tissue resection has witnessed dramatic changes over the last four decades. It started in the form of endoscopic mucosal resection (EMR) in the early 1980s and, subsequently, endoscopic submucosal dissection (ESD) came into play which has now proven itself as a very popular means of resecting EGCs [7].

In accordance with the Japanese guidelines, patients with EGC without lymph node involvement are given specific criteria for diagnosis [8, 9]. To that end, absolute indication is considered in patients with EGCs which: are less than 2 cm in size, have differentiated morphology with intra-mucosal location, and are devoid of ulcer. On the other hand, indications are expanded for early stage gastric lesions which: measure greater than 2 cm, hardly extend beyond the mucosal layer, have differentiated histology and lack ulceration or if size is less than 3 cm in presence of ulceration, and undifferentiated lesions where size is less than 2 cm. Lympho-vascular involvement, however, should not be present in both absolute and expanded indications [8, 9]. The above-mentioned indications were established after evaluating the likelihood of metastasis to lymph nodes in specimens obtained from gastrectomy [10].

There is a general consensus that during selection of patients with superficial gastric neoplasia for endoscopic treatment, complete removal of a lesion has to be assured with no lymph nodes involved and the margins’ status has to be clear [3, 11]. However, the definition applied for EGCs at the present fails to take account of lymph node status [12–14] and, hence, nothing is mentioned about lymph node involvement after microscopic evaluation of specimens obtained from endoscopic resection [15, 16]. To make matters worse, there is no any single imaging modality to date that can help experts to preoperatively decide on lymph node status with full confidence [17–19]. Yet, metastasis to lymph nodes is seen in patients with EGCs that varies according to the extent of invasion (intra-mucosal vs submucosal) and the risk is lower in the former (less than 3%) as compared to the latter (up to 20%) [10, 20–22]. If regional lymph nodes remain involved after endoscopic resection, it implies that the endoscopic treatment is suboptimal and patients would end up having additional interventions on top of ESD [23]. The ultimate goal of ESD, nevertheless, is to attain curability which can be assessed by microscopic evaluation of resected specimens using a set of inclusion criteria (absolute and expanded) as stated above [9]. And the post-ESD clinical results are similar for all patients not exceeding the expanded criteria as could be indicated by many studies conducted in the Asian continent [24–28].

It is worthy to adopt an *en*-bloc means of resection for EGCs in order to obtain a specimen that has appropriate histologic details for reliable assessment and potential reduction in the emergence of recurrent lesions [3, 29]. The attainment of complete resection is declared when an *en*-bloc resected lesion lacks both margin positivity and lympho-vascular invasion. Following ESD procedure, patients who do not conform to the inclusion criteria are assumed to have non-e-curative (NEC) outcomes and may require further intervention depending on certain cicumstances such as fear of metastasis to lymph nodes as well as for having bad prognostic behavior [10, 13]. Nonetheless, the risk of lymph node metastasis in patients who received NEC resection treatment after undergoing gastrectomy was found to be less than 10% [3, 30–32]. In addition, there is a growing number of elderly patients with or without concomitant illnesses who are unfit for operation [33]. Hence, the need to identify tumor-associated factors which determine oncologic outcome in ESD is indispensable in order to have appropriate selection of candidates for subsequent surgical intervention. Of note, there is an ever-changing trend in both technical aspects as well as indications for patients with early gastric neoplasia in the setting of ESD. Thus, the goal of our study was to investigate the potential clinico-pathologic parameters that lead to NEC endoscopic resection following ESD. Moreover, we performed analysis of various tumor-associated factors to unveil their role in submucosal invasion in patients with EGC.

## Methods

### Inclusion and exclusion criteria

**Fig. 1:**
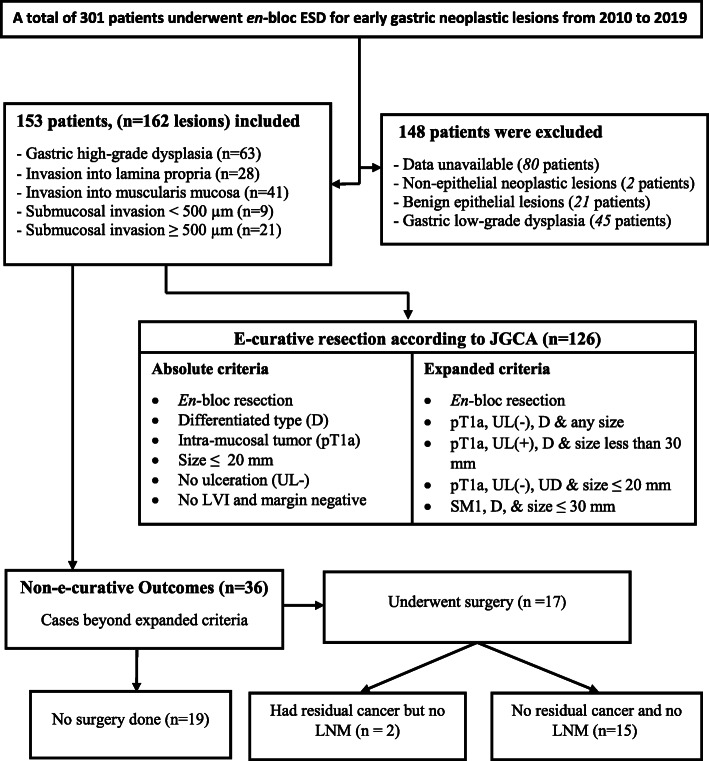
Flowchart for patients with early gastric neoplasia who underwent ESD. ESD = Endoscopic submucosal Dissection, EGC = early gastric cancer, JGCA = Japanese Gastric Cancer Association, UD = Undifferentiated, U = Ulcer, SM1= Submucosal invasion < 500 μm LVI = Lympho-vascular invasion, UL= Ulceration, LNM = lymph node metastasis type

Figure 1, illustrates the general enrolment process of patients with early gastric neoplasia into our study. Accordingly, there were a total of 301 patients who received *en*-bloc ESD after fulfilling absolute or expanded indications. Briefly, we excluded 148 patients who had the following features: detailed data were unavailable (*n*=80), neoplastic lesions with non-epithelial origin (*n*=2), non-neoplastic epithelial lesions (*n*=21), and gastric low-grade dysplasia (*n*=45). Notably, we did not consider those patients who underwent endoscopic mucosal resection or piecemeal type of resection. After exclusion of the above-mentioned cases, a total of 162 early gastric neoplastic lesions (from 153 patients) were retained for subsequent descriptive, comparative and logistic regression analysis. In this study, we made analyses of risk factors based on individual neoplastic lesions as some of the study subjects had multiple tumors resected by *en*-bloc ESD.

### ESD procedure and specimen preparation

All enrolled patients underwent proper endoscopic evaluation, and ESD procedure was in turn performed by experienced endoscopist. The types of ESD knives and electrocautery used were DualKnife (KD-650 L; Olympus, Tokyo, Japan), ITknife2 (KD-611 L; Olympus), Coagrasper (FD-410LR; Olympus), and Gastroscope (GIF-Q260J; Olympus). The margin of resection was marked using electrocoagulation knife with inclusion of nearly 0.5 cm normal mucosal tissue around the lesion. A solution of normal saline was injected into the submucosa to elevate the lesion off the muscularis propria. The mucosa and submucosa on the outer edge of the lesion were incised circumferentially along the previously marked margin. Then, the submucosa was dissected until the mucosal lesion had been completely resected. Preventive coagulation was implemented for exposed blood vessels in the post-ESD resection defects. The measurements of the resected lesion and mucosal defects were recorded. The resected specimens were immediately stretched and pinned on a flat polystyrene board to prevent folding, and fixed in 10% formalin to facilitate optimal orientation during paraffin embedding. For histologic evaluation, the fixed specimens were serially sectioned at 2-mm intervals.

### Study design and period

The present study is retrospective in design which involved patients diagnosed as early gastric epithelial neoplasia on whom ESD resection was consecutively performed at a tertiary hospital (Tongji Hospital, Wuhan, China). The study period ranges from January 2010 to October 2019.

### Study setting

This study was conducted in the Institute of Pathology at Tongji Hospital, Tongji Medical College of Huazhong University of Science and Technology, Wuhan, China.

### Data collection

The demographic information of patients including age and gender was obtained from previous medical records. Similarly, data related to endoscopic tumor characteristics such as macroscopic type, location, tumor size, and ulcerative findings were gleaned from previous records of endoscopic examination done by experts in the field. The processed tissue specimens in the form of slides were searched in an archive according to the identification number of each recruited patient in its respective shelf. After collecting all the slides, we arranged them serially in accordance with the corresponding year of ESD resection. After that, we began microscopic evaluation of all the slides and data pertaining to each parameter were recorded.

For difficult cases, proper consultation to senior experts was done to re-evaluate cases under multi-headed microscope to avoid discordance in the final histopathologic assessment. A new slide preparation was requested from reserved tissue blocks (formalin fixed paraffin embedded) as needed, especially for older slides in which case evaluation was difficult owing to poor slide quality. In such instances, a thin slice of specimen (4 μm thick) was prepared using a sectioning microtome, and routine hematoxylin and eosin staining was carried out followed by microscopic evaluation of slides. We made meticulous histopathologic evaluation of ESD-resected specimens with necessary discussions and consultations to minimize intra-observer bias.

### Definitions and clinico-pathologic assessment

On the whole, 153 patients with early gastric neoplasia were recruited into our study, all of whom received an *en*-bloc type ESD based on the indications established by JGCA [3, 9]. However, subsequent histopathologic evaluation of all lesions was performed by adopting the JGCA guideline established in 2014 [9]. Accordingly, the lesions were grouped as e-curative and NEC based on their respective clinical and pathological characteristics. As per the JGCA [9] (Fig. 1), curative resection is declared for EGCs that conform to both absolute and expanded criteria after ESD procedure. Patients with absolute indication should have a differentiated morphology, size of two centimeters or below, and tumors have to be confined to the mucosal region. Additionally, curative resection is considered for patients with expanded indications that include: 1) differentiated intra-mucosal tumor of any size that has no ulceration, 2) ulcerated intra-mucosal cancer with differentiated histology having maximum diameter of ≤ 30 mm, 3) tumor size of ≤ 30 mm with submucosal invasion of < 500 μm as measured from the muscularis mucosa, 4) an intra-mucosal tumor of ≤ 20 mm diameter with undifferentiated histology and is devoid of ulcer. On top of satisfying the absolute and expanded indications, the type of resection has to be *en*-bloc, both horizontal and vertical margins negative, and lympho-vascular involvement should also be negative. The NEC group are those who do not fulfill the above-mentioned criteria.

**Fig. 2:**
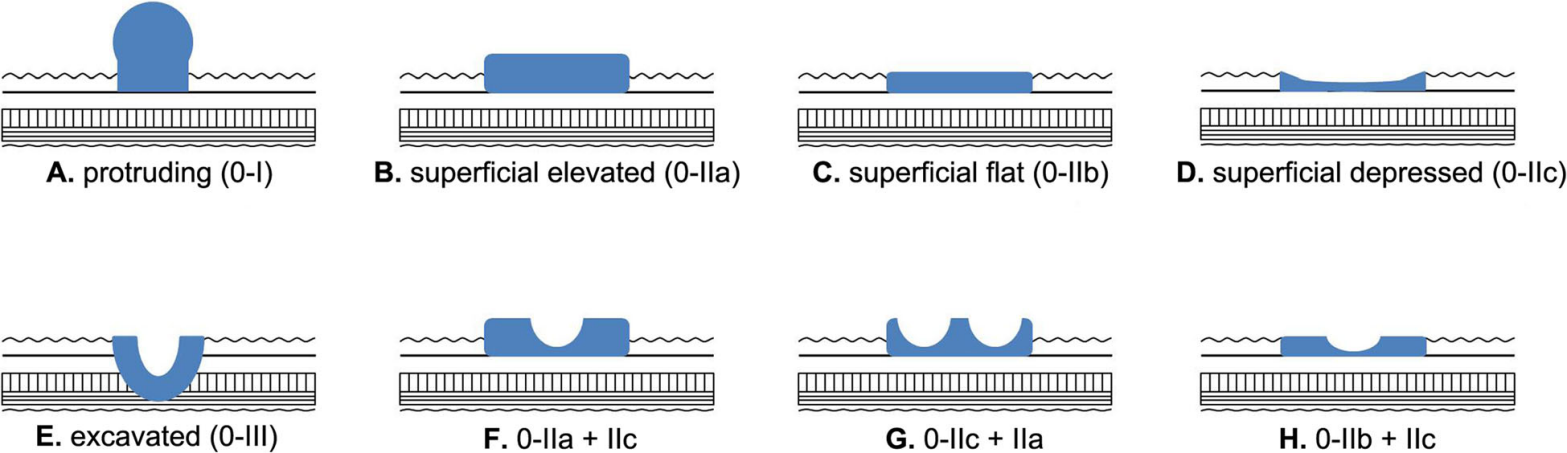
Endoscopy-based macroscopic sub-classification of type ‘0’ early gastric lesions. **A)** Protruding [0-I], with ≥ 3 mm elevation. **B)** Superficial elevated [0-IIa], with < 3 mm elevation. **C)** Superficial flat lesions [0-IIb], tumors with no elevation or depression. **D)** Superficial depressed [0-IIc], tumors with slight depression. **E)** Excavated [0-III], tumors which are deeply depressed. **F)** Superficial elevated and depressed lesions with predominant elevated type [0-IIa + IIc]. **G)** Superficial elevated and depressed lesions with predominant superficial depressed type [0-IIc + IIa]. **H)** Superficial flat and depressed lesions with predominant superficial flat type [0-IIb + IIc].

Three intra-gastric tumor locations namely upper, middle and lower one-thirds were considered during our analysis. Tumors seen during endoscopic inspection were classified based on macroscopic features as: protruding (0-I), purely superficial elevated (0-IIa) or mixed type with predominant superficial elevated, purely superficial flat (0-IIb) or mixed with predominant superficial flat type and purely superficial depressed (0-IIc) or mixed with predominant superficial depressed types (see Fig. 2). There were no lesions belonging to excavated (0-III) macroscopic variant in this study. For the purpose of analysis, tumors were divided into two groups with respect to their macroscopic type. ‘Group 1’ constituted protruding (0-I), purely superficial flat (0-IIb) and mixed variants of superficial flat lesions, and ‘Group 2’ included purely superficial elevated or depressed lesions (0-IIa or IIc) and/or their corresponding mixed variants where the superficial elevated or depressed is a predominant feature. The maximum diameter of each lesion (in centimeter) was taken into account during microscopic visualization, and ulceration status was thoroughly evaluated with dichotomous result (absence vs presence). Diagnostic decision on ulcerative findings was dependent on histologic evaluation of a tissue but information from endoscopic examination was also incorporated to provide a final conclusion. Further evaluation of the remaining parameters depended on histopathological examination of the ESD-resected specimen. The degree of histologic tumor differentiation was divided as differentiated (D) type in which case a well or moderately differentiated tubular or papillary adenocarcinoma is identified. On the contrary, if a tumor showed poor differentiation or a signet ring cell type of carcinoma or mucinous type adenocarcinoma, it was regarded as having undifferentiated (UD) histology. If a single tumor lesion displays both histologic variants of differentiated and undifferentiated histology, the type that is quantitatively predominant would be considered.

**Fig. 3:**
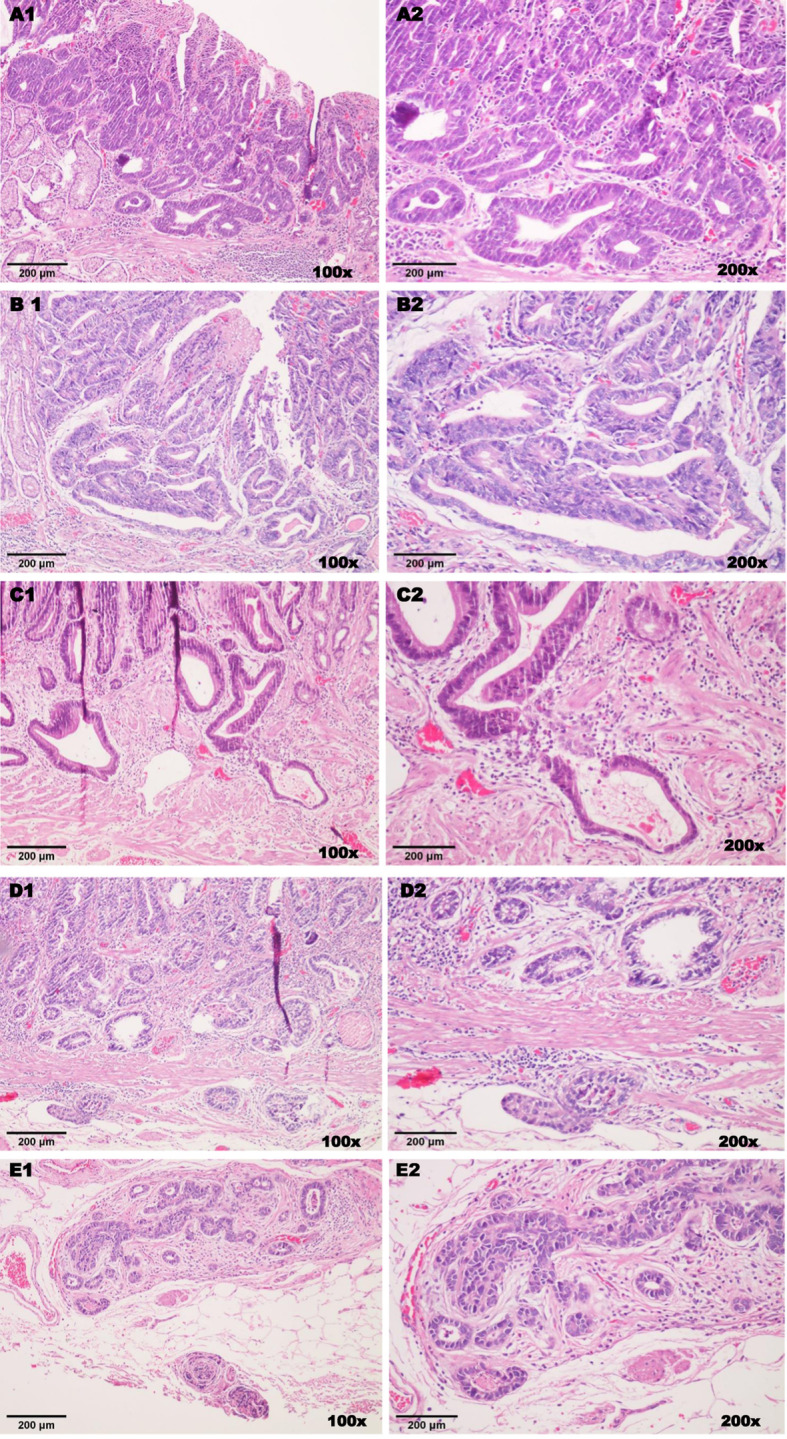
Different stages of early gastric neoplasia according to the depth of invasion (total magnification = 100x & 200x, scale = 100 & 200 μm). Hematoxylin and Eosin staining. (**A**1/**A**2) Gastric high grade dysplasia displaying complex glandular structure, significant cellular atypia, nuclear pseudo-stratification, hyperchromasia and poor nuclear polarization. (**B**1/**B**2) Cancer invading lamina propria. (**C**1/**C**2) Invasion involving the muscularis mucosae layer. (**D**1/**D**2) Gastric cancer that infiltrates into the submucosal layer but less than 500 μm depth from the muscularis mucosae, there is also associated lymphatic invasion. (**E**1/**E**2) Submucosal invasion greater than 500 μm from the muscularis mucosae with positive vertical margin

The Lauren’s classification scheme was also used to classify EGCs as intestinal, diffuse and mixed. The extent of tumor invasion for the superficial gastric lesions was assessed by taking the muscularis mucosa as landmark (shown in Fig. 3). Hence, there were three groups of patients in relation to tumor invasion as follows: in one group, the tumor is confined to the mucosa (M), in the second group, invasion extends deep into the submucosa but hardly exceeds 500 μm as measured from the muscularis mucosa (SM1), and the last set included patients with greater or equal to 500 μm submucosal penetration from the muscularis mucosa (SM2). But during our analyses, we grouped patients into two as mucosal and submucosal (SM1 and SM2) with respect to the depth of tumor invasion as the number of lesions with submucosal invasion was low. In addition, we assessed the lymphatic and vascular invasion characteristics, where the expected outcome fell into either absent or present. Furthermore, we evaluated margin clearance status (both horizontal and vertical margins) with end results falling into two-tier class as positive or negative.

### Statistical analysis

Relevant data was initially entered into an Excel Spreadsheet and then imported into Statistical Package for the Social Sciences version 23 for final analysis. While executing descriptive analysis, continuous variables were calculated as mean ± standard deviation, and categorical variables were expressed using frequency and percentages. A Chi-squared test or Fisher’s exact probability test (as appropriate) and student’s *t*-test were used to investigate possible differences among categorical and continuous variables, respectively. In an attempt to investigate whether tumor-related factors had predictive effect on curability and depth of tumor invasion, a logistic regression model was applied. To that end, each variable was first tested using univariate analysis. Subsequently, all the variables that showed significance in the univariate analysis were retained for multivariate analysis in order to sort out the factors that were independently associated with NEC resection and submucosal infiltration after ESD. Two-tailed *p* < 0.05 was considered statistically significant. Calculation of both odds ratios (OR) and the 95% confidence intervals (95% CI) were made in order to estimate the relative risk of NEC outcomes and depth of submucosal invasion following ESD resection as well as to measure the degree of association among different clinico-pathologic factors.

## Results

### Baseline characteristics of study patients following ESD procedure

A total of 162 early gastric neoplastic lesions (153 patients) received an *en*-bloc type of ESD resection. Table 1 provides a summarized baseline information of recruited patients including frequency distribution and their corresponding clinical parameters. The rate of completeness of resection and NEC outcome were 95% and 22.2%, respectively. Three fourth of the participants (74.1%) were males and the mean age (± standard deviation) of the study population was 61.10 ± 8.76 years.

**Table 1:** Overall frequency and percentage distribution of patients with early gastric neoplasia after ESD procedure

Clinico-pathologic parameter	Total number of EGC lesions (***n***=162)	Percentage
**Mean Age in years (±SD)**	61.10 ± 8.76	–
**Gender**		
Female	42	25.9%
Male	120	74.1%
**Tumor location**		
Lower one third	95	58.6%
Middle or Upper third	67	41.4%
**Gross tumor type**		
Group 1	46	28.4%
Group 2	116	71.6%
**Ulceration status**		
Absent	135	83.%
Present	27	16.7%
**Size of tumor**		
≤ 2 cm	111	68.5%
> 2 cm	51	31.5%
**Differentiation**		
Differentiated	129	79.6%
Undifferentiated	33	20.4%
**Lauren’s category**		
Intestinal	129	79.6%
Diffuse/Mixed	33	20.4%
**Level of tumor invasion**		
Intra-mucosal tumor	132	81.5%
Submucosal tumor	30	18.5%
**Horizontal margin**		
Negative	159	98.1%
Positive	3	1.9%
**Vertical margin**		
Negative	158	97.5%
Positive	4	2.5%
**Lympho-vascular invasion**		
Absent	159	98.1%
Present	3	1.9%
**Resection type (*en*-bloc)**	162	100%
**Curability outcome (non-e-curative)**	36	22.2%
**Complete resection**	154	95%

*ESD* Endoscopic Submucosal Dissection, *EGC* Early Gastric Cancer, *SD* Standard Deviation, *Group 1* = lesions with Protruding (0-I), Purely superficial flat (0-IIb) and Mixed variants with 0-IIb predominant, *Group2* = Purely superficial elevated (0-IIa) or depressed (0-IIc) or mixed variants with either 0-IIa or 0-IIc predominant

Majority of the neoplastic lesions (58.6%) were located in the lower one third of the stomach and 71.6% of all the tumors had a macroscopic type belonging to the group with purely superficial elevated or depressed lesions (0-IIa or 0-IIc) and/or their corresponding mixed variants where the superficial elevated or depressed is a predominant feature (Group 2). All other macroscopic lesions such as protruding (0-I), purely superficial flat (0-IIb) and mixed variants of superficial flat constituted for 28.4% (Group 1). A large proportion of gastric lesions had a size ≤ 2 cm (68.5%, 111 lesions), absence of ulceration (83.0%, 135 lesions), and differentiated type of histology (79.6%, 129 lesions). According to Lauren’s classification of gastric tumors, a total of 129 lesions (79.6%) displayed an intestinal-type morphology whereas nearly 20% were non-intestinal (diffuse/mixed histology). In terms of depth of tumor invasion, there were higher number of intra-mucosal lesions as compared to the submucosa, 81.5% and 18.5%, respectively. The horizontal margin was positive in three lesions (1.9%), and four lesions (2.5%) had positive vertical margin. Positive lympho-vascular invasion was identified in three lesions (1.9%).

The baseline characteristics of patients with regard to depth of tumor invasion (mucosal vs submucosal) are shown in Table 4. Out of 162 lesions, 18.5% (30 lesions) showed invasion into the submucosal layer. Considering tumor location and macroscopic endoscopic features, tumors in the upper two third of the stomach and those having 0-IIa/0-IIc or their mixed types with 0-IIa/0-IIc predominant had submucosal invasion of 80% and 90%, correspondingly. In terms of histologic differentiation, the number of cases in both differentiated and undifferentiated groups exhibiting submucosal invasion were equal (50% each).

### Factors that contributed to NEC resection after ESD procedure

Table 2 summarizes the demographic and clinico-pathologic factors of patients with early gastric neoplasia in relation to the post-ESD curability outcome. Accordingly, all patients were divided into two groups (e-curative vs non-e-curative). Overall, there were 126 early gastric lesions (77.8%) that received curative ESD resection whereas 36 lesions had a NEC resection (22.2%). We tried to compare e-curative and NEC groups in terms of their demographic and clinico-pathologic features using a Chi-squared independent test or Fisher’s exact test and student *t*-test applied as appropriate. Hence, there was a significant difference in curability outcome among tumor-associated factors such as tumor location, differentiation, size of tumor, and depth of tumor invasion (all with *p* < 0.001). Likewise, a significant difference was found between these groups of patients with regard to macroscopic tumor type (*p* = 0.002), ulcerative finding (*p* = 0.011), horizontal margin (*p* = 0.010), vertical margin (*p* = 0.002) and lympho-vascular invasion (*p* = 0.010) following endoscopic resection. On the other hand, no significant difference was observed with respect to gender of patients (*p* = 0.472) and the difference in age was only marginally significant (*p* = 0.046).

**Table 2:** Baseline characteristics of patients and their difference in curability outcomes following ESD

Patient Parameter	All EGCs lesions (***n*** = 162) [No. (%)]	E-curative (***n*** = 126) [No. (%)]	Non-e-curative (***n*** = 36) [No. (%)]	p*-value
**Mean age in years (±SD)**	61.10 (± 8.76)	60.37 (± 8.27)	63.67 (± 10.0)	**0.046**
**Gender**				0.472
Female	42 (25.9)	31 (24.6)	11 (30.6)	
Male	120 (74.1)	95 (75.4)	25 (69.4)	
**Tumor location**				**< 0.001**
Lower third	95 (58.6)	84 (66.7)	11 (30.6)	
Middle or Upper third	67 (41.4)	42 (33.3)	25 (69.4)	
**Gross tumor type**				**0.002**
Group 1	46 (28.4)	43 (34.1)	3 (8.3)	
Group 2	116 (71.6)	83 (65.9)	33 (91.7)	
**Ulceration status**				**0.011**
Absent	135 (83.3)	110 (87.3)	25 (69.4)	
Present	27 (16.7)	16 (12.7)	11 (30.6)	
**Size of tumor**				**< 0.001**
≤ 2 cm	111 (68.5)	98 (77.8)	13 (36.1)	
> 2 cm	51 (31.5)	28 (22.2)	23 (63.9)	
**Differentiation**				**< 0.001**
Differentiated	129 (79.6)	115 (91.3)	14 (38.9)	
Undifferentiated	33 (20.4)	12 (9.5)	21 (60.0)	
**Lauren’s category**				< 0.001
Intestinal	129 (79.6)	115 (91.3)	14 (38.9)	
Diffuse/ Mixed	33 (20.4)	11 (8.7)	22 (61.1)	
**Level of tumor invasion**				**< 0.001**
Intra-mucosal tumor	132 (81.5)	124 (98.4)	8 (22.2)	
Submucosal tumor	30 (18.5)	2 (1.6)	28 (77.8)	
**Horizontal margin**				**0.010**
Negative	159 (98.1)	126 (100)	33 (91.7)	
Positive	3 (1.9)	0 (0.0)	3 (8.3)	
**Vertical margin**				**0.002**
Negative	158 (97.5)	126 (100)	32 (88.9)	
Positive	4 (2.5)	0 (0.0)	4 (11.1)	
**Lympho-vascular invasion**				**0.010**
Absent	159 (98.1)	126 (100)	33 (91.7)	
Present	3 (1.9)	0 (0.0)	3 (8.3)	

*ESD* Endoscopic Submucosal Dissection, *EGC* Early Gastric Cancers, *SD* Standard Deviation, *Group 1* = lesions with Protruding (0-I), Purely superficial flat (0-IIb) and Mixed variants with 0-IIb predominant, *Group2* = Purely superficial elevated (0-IIa) or depressed (0-IIc) or mixed variants with either 0-IIa or 0-IIc predominant.

*Chi-squared independent test/Fisher’s exact test of probability or Student t-test as appropriate

The predictive impact of all clinico-pathologic factors on patients with early gastric cancer following ESD procedure was investigated using logistic regression analysis as depicted in Table 3.

**Table 3:** Factors that contributed to non-e-curative outcome in patients with early gastric cancers after ESD

Clinico-pathologic parameters	Univariate analysis		Multivariate analysis	
	OR (95% CI)	***p-***value	OR (95% CI)	***p-***value
**Age (in years)**	1.05 (1.00–1.09)	**0.049**	1.08 (1.01–1.17)	**0.029**
**Tumor location**				
Lower one third	1 (Reference)		1 (Reference)	
Middle or Upper third	4.55 (2.04–10.12)	**< 0.001**	5.46 (1.65–18.12)	**0.006**
**Gross tumor type**				
Group 1	1 (Reference)		1 (Reference)	
Group 2	5.70 (1.65–19.65)	**0.006**	9.77 (1.23–77.65)	**0.031**
**Ulceration status**				
Absent	1 (Reference)		1 (Reference)	
Present	3.03 (1.25–7.31)	**0.014**	2.39 (0.59–9.65)	0.223
**Size of tumor**				
≤ 2 cm	1 (Reference)		1 (Reference)	
> 2 cm	6.19 (2.79–13.77)	**< 0.001**	7.63 (2.29–25.42)	**0.001**
**Differentiation**				
Differentiated	1 (Reference)		1 (Reference)	
Undifferentiated	16.43 (6.60–40.89)	**< 0.001**	15.54 (1.65–146.22)	**0.016**
**Lauren’s category**				
Intestinal	1 (Reference)		1 (Reference)	
Non-intestinal	16.43 (6.60–40.89)	**< 0.001**	3.29 (0.36–31.40)	0.300

*ESD* Endoscopic Submucosal Dissection, *OR* Odds Ratio, *CI* Confidence Interval, *Group 1* = lesions with Protruding (0-I), Purely superficial flat (0-IIb) and Mixed variants with 0-IIb predominant, *Group2* = Purely superficial elevated (0-IIa) or depressed (0-IIc) or mixed variants with either 0-IIa or 0-IIc predominant

Initially, the role of each tumor-associated variable was examined separately for curability outcome in a univariate analysis. As a result, significant effect on non-e-curability was obtained in association with tumors located in the upper two third of the stomach, larger tumor size (> 2 cm), undifferentiated histology and tumors that belonged to diffuse or mixed by Lauren’s classification (all with *p* < 0.001). In addition, univariate analysis revealed significant associations with NEC resection when lesions: were ulcerated (*p* = 0.014), had purely superficial elevated or depressed or their mixed counterparts (*p* = 0.006), and when patients had advanced age (*p* = 0.049).

Finally, a multivariate logistic regression analysis was carried out (Table 3) in which all the clinico-pathologic factors were considered together in order to further investigate factors that independently predict a NEC outcome. Thus, tumor location in the upper two third of the stomach (odds ratio [OR], 5.46; 95% confidence interval [95% CI], 1.65–18.12; *p* = 0.006), tumor size greater than 2 cm (OR, 7.63; 95% CI, 2.29–25.42; *p* = 0.001), microscopically undifferentiated tumor (OR, 15.54; 95% CI, 1.65–146.22; *p* = 0.001), and old age (OR, 1.08; 95% CI, 1.01–1.17; *p* = 0.029) were found to have independent predictive influence on NEC resection of early gastric tumors.

In a similar fashion, purely superficial elevated or depressed or mixed gross type lesions with either elevated or depressed component predominating, independently contributed to a NEC outcome (OR, 9.77; 95% CI, 1.23–77.65; *p* = 0.031). The presence of ulceration (OR, 2.39; 95% CI, 0.59–9.65; *p* = 0.223) and non-intestinal (diffuse or mixed) tumors by Lauren’s classification (OR, 3.29; 95% CI, 0.36–31.40; *p* = 0.300) did not show any significant contribution to no-curability after multivariate analysis.

### Factors associated with submucosal invasion in patients with EGC after ESD

Herein, we conducted both comparative and multiple logistic regression analysis of clinico-pathologic factors with respect to the depth of invasion as illustrated in Table 4 and Table 5, respectively. A remarkably significant difference was obtained in tumors located in the upper two third vs lower third of the stomach (*p* < 0.001), macroscopic type of 0-IIa/0-IIc or their mixed types with 0-IIa/0-IIc predominant gross type vs 0-I/0-IIb and mixed with 0-IIb dominant (*p* = 0.013), lesions with ulcerative finding vs non-ulcerated (*p* = 0.001), size ≥ 2 cm vs size < 2 cm (*p* = 0.001), and undifferentiated vs differentiated histology (*p* < 0.001).

**Table 4:** Baseline characteristics of patients with EGC and their difference in terms of depth of invasion following ESD procedure

Patient Parameters	All EGCs lesions (***n*** = 162) [No. (%)]	Mucosal lesion (***n*** = 132) [No. (%)]	Submucosal (***n*** = 30) [No. (%)]	p*-value
**Mean age in years (±SD)**	61.10 (± 8.76)	60.33 (± 8.38)	64.53 (± 9.70)	0.563
**Gender**				0.981
Female	42 (25.9)	34 (25.8)	8 (26.7)	
Male	120 (74.1)	98 (74.2)	22 (73.3)	
**Tumor location**				**< 0.001**
Lower third	95 (58.6)	89 (67.4)	6 (20.0)	
Middle or Upper third	67 (41.4)	43 (32.6)	24 (80.0)	
**Gross tumor type**				**0.013**
Group 1	46 (28.4)	43 (32.6)	3 (10.0)	
Group 2	116 (71.6)	89 (67.4)	27 (90.0)	
**Ulceration status**				**0.001**
Absent	135 (83.3)	116 (87.9)	19 (63.3)	
Present	27 (16.7)	16 (12.1)	11 (36.7)	
**Size of tumor in**				**0.001**
≤2 cm	111 (68.5)	98 (74.2)	13 (43.3)	
> 2 cm	51 (31.5)	34 (25.8)	17 (56.7)	
**Differentiation**				**< 0.001**
Differentiated	129 (79.6)	114 (86.4)	15 (50.0)	
Undifferentiated	33 (20.4)	18 (13.6)	15 (50.0)	
**Horizontal margin**				0.088
Negative	159 (98.1)	131 (99.2)	28 (91.7)	
Positive	3 (1.9)	1 (0.8)	2 (8.3)	
**Vertical margin**				**0.001**
Negative	158 (97.5)	132 (100)	26 (86.7)	
Positive	4 (2.5)	0 (0.0)	4 (13.3)	
**Lympho-vascular invasion**				**< 0.001**
Absent	159 (98.1)	132 (100)	27 (90.0)	
Present	3 (1.9)	0 (0.0)	3 (10.0)	

*ESD* Endoscopic Submucosal Dissection, *EGC* Early Gastric Cancers, *Group 1* = lesions with Protruding (0-I), Purely superficial flat (0-IIb) and Mixed variants with 0-IIb predominant, *Group 2* = Purely superficial elevated (0-IIa) or depressed (0-IIc) or mixed variants with either 0-IIa or 0-IIc predominant

*Chi-squared independent test/Fisher’s exact test of probability or Student t-test

**Table 5:** Risk factors for submucosal invasion in patients with early gastric cancer following Endoscopic Submucosal Dissection

Clinico-pathologic parameters	Univariate analysis		Multivariate analysis	
	OR (95% CI)	***p-***value	OR (95% CI)	***p-***value
**Tumor location**				
Lower one third	1 (Reference)		1 (Reference)	
Middle or Upper third	8.28 (3.15–21.75)	**< 0.001**	8.88 (2.90–27.17)	**< 0.001**
**Gross tumor type**				
Group 1	1 (Reference)		1 (Reference)	
Group 2	4.35 (1.25–15.13)	**0.021**	3.59 (0.84–15.45)	0.086
**Ulceration status**				
Absent	1 (Reference)		1 (Reference)	
Present	4.20 (1.69–10.41)	**0.002**	3.70 (1.15–11.90)	**0.028**
**Size of tumor**				
≤ 2 cm	1 (Reference)		1 (Reference)	
> 2 cm	3.77 (1.66–8.57)	**0.002**	2.94 (1.08–8.02)	**0.036**
**Differentiation**				
Differentiated	1 (Reference)		1 (Reference)	
Undifferentiated	6.33 (2.65–15.14)	**< 0.001**	6.51 (2.23–18.98)	**0.001**

*OR* Odds Ratio, *CI* Confidence Interval, *Group 1* = lesions with Protruding (0-I), Purely superficial flat (0-IIb) and Mixed variants with 0-IIb predominant, *Group2* = Purely superficial elevated (0-IIa) or depressed (0-IIc) or mixed variants with either 0-IIa or 0-IIc predominant

Later on, univariate and multivariate logistic regression analyses were carried out on the factors where a statistically significant difference was observed (Table 5). In the univariate analysis, submucosal infiltration was significantly related with tumors of upper two third location, gross type 0-IIa/0-IIc or their mixed types with 0-IIa/0-IIc predominant, ulcerated lesions, lesions with ≥ 2 cm size and undifferentiated histology (all with *p* < 0.05). Multivariate logistic regression analysis revealed that the following tumor-related factors were independent predictors of submucosal invasion: location in the upper two third of the stomach (OR, 8.88; 95% CI, 2.90–27.17; *p* < 0.001), ulcerated lesions (OR, 3.70; 95% CI, 1.15–11.90; *p* = 0.028), lesions with greater than 2 cm (OR, 2.94; 95% CI, 1.08–8.02; *p* = 0.036) and those with poor differentiation (OR, 6.51; 95% CI, 2.23–18.98; *p* = 0.001).

### Results of gastrectomy specimens for patients who had NEC outcome following ESD

Parallel to evaluation of ESD resected specimens, we looked at some of the gastrectomy specimens together with their lymph node dissections for patients who had a NEC with an attempt to determine the presence of residual tumor or/and lymph node metastasis (Fig. 1). Hence, out of the total 36 lesions which fulfilled the criteria for NEC outcome, 17 patients received additional surgical treatment. Fifteen of those patients who received additional surgery showed evidence of mucosal defect with chronic ulcerative and inflammatory changes or/and granulation tissue. But no cancerous cells were detected in the resected lymph nodes and surrounding tissues. Residual cancer was found in two patients who underwent surgery but none of them exhibited metastatic disease in the dissected lymph nodes.

## Discussion

A timely detection of early gastric cancers and appropriate selection of their means of treatment is very crucial as it results in favorable prognostication and reduced mortalities [11]. A remarkably high rate (95%) of 5-year survival has been reported in patients with early gastric cancer who underwent ESD fulfilling the indications for expanded criteria [34, 35]. Despite the curative intent of ESD procedure, there is an inevitable NEC resection in about 11.9%–21.4% of EGC patients who might require additional surgical treatment depending on their overall situation [36–41]. Unlike those tumor-associated factors such as location, size and macroscopic type, it is difficult to accurately predict the depth of tumor invasion and lympho-vascular involvement using endoscopy-based pre-ESD evaluations [42]. Recent studies indicated that conventional endoscopic methods had about 72%–78% accuracy in predicting the level of tumor infiltration in EGCs. Likewise, a similar precision rate (67% to 85%) in extent of invasion has been reported for ultra-sonographic endoscopy, which is a common method of evaluation in several institutions [17–19].

Whatsoever the case, appropriate selection of candidates for subsequent additional surgery is essential in the setting of non-curability following ESD resection. This is in order to obviate the possibility of local recurrence and metastasis to lymph nodes that are potentially associated with NEC endoscopic resection. Additionally, it helps to eliminate unnecessary ESD procedure on patients if they finally have to end up doing surgery following NEC outcome. The present study indicated that the rate of complete resection and non-curability were 95% and 22.2%, respectively, which is in harmony with several previous reports [36–41]. Of note, our study identified 36 patients who had NEC resection because their conditions went beyond the absolute and expanded criteria following *en*-bloc ESD (Fig. 1). Other factors such as involvement of lympho-vascular invasion and positive margin status (horizontal or/and vertical margins) were the reasons for non-curative resection in these patients.

The utilization of ESD for management of early gastric lesions has attained tremendous popularity and the need for expansion of the indication criteria for resection is also increasing. Therefore, sufficient and reliable data on factors that are associated with non-curability following endoscopy-based gastric tumor resection is vital for better clinical outcomes. In terms of respectability, the *en*-bloc means of resection provides a better curative outcome as compared to a piecemeal method [43]. Therefore, in this particular study, we aimed to investigate the demographic and tumor-associated parameters that predict submucosal infiltration and a NEC outcome in patients with early gastric neoplasia who received *en*-bloc ESD resection.

Overall, the results of our multivariate analysis indicated that the following factors were independently associated with both submucosal invasion and NEC outcomes: 1) upper tumor location (upper two third of the stomach), 2) larger tumor size (> 2 cm), and 3) tumors with undifferentiated histologic pattern. Tumors with purely superficial elevated (0-IIa) or depressed (0-IIc) or mixed gross type with predominant superficial elevated or depressed component (mixed with 0-IIa or 0-IIc dominant) and advanced age were also identified as independent predictors of non-e-curability. In addition to that, ulcerated lesions showed significant association with submucosal infiltration.

Many studies have shown that upper tumor location, enlarged tumor size and undifferentiated microscopic feature independently predict for NEC outcomes, which is consistent with the present study [39, 40, 44–46]. Others reported that tumors located in the upper two third of stomach had higher rate of submucosal invasion and hence a greater frequency of NEC resection as opposed to tumors of the lower third (antrum), which is in line with our study [47, 48]. Similarly, it was clearly described that proximally located gastric tumors behave more aggressively and have a worse prognosis compared to distal counterparts [48–53]. EGCs located in the middle and upper third are not detected as early, owing to the technical difficulty faced during endoscopic evaluation and thus compromising diagnostic yield from forceps biopsy. This is because maintaining a front view of endoscopy is challenging and it has to be used in its retroflexed fashion [54]. Additionally, there is a difference in thickness of submucosal layer between body and antrum, with the former being thinner as compared to the latter [55]. Last but not least, relatively abundant lymphatic vessels are found in the lamina propria of cardia region as opposed to lower regions of the stomach [56]. By combining all these evidences, EGCs situated in the upper portion of the stomach are likely to result in NEC resection and hence, special caution is needed while dealing with tumors in this location.

The real impact of tumor size on ESD outcome has been debated by many researchers. One large-scale study [24] indicated that the size of tumor has nothing to do with curability outcome after ESD. On the contrary, the present study has shown a significant contribution of larger tumor size (> 2 cm) to NEC outcome in a multivariate analysis. In line with our study, Imagawa et al. reported a significant difference (*p* < 0.0001) in curability outcome between lesions of ≤ 2 cm and > 2 cm, 59% vs 89%, respectively. Lee et al. indicated that there was a significant correlation between depth of tumor invasion and its size (*p* < 0.001). As a rule of thumb, the bigger the lesion is, the more extensive the vascular bed will be, and excess bleeding is anticipated that might interfere with ESD procedure. Moreover, data on a large series of cases [10] demonstrated that tumors with larger than 3 cm are significantly correlated with an increased risk of metastasis to lymph nodes. For the aforementioned reasons, the post-ESD outcome is likely to be influenced by large size EGCs.

Our study demonstrated that EGCs with undifferentiated histology are significantly associated with NEC outcome in a multivariate analysis. The morphologic patterns of both differentiated and undifferentiated gastric adenocarcinoma are somewhat dissimilar. According to several recent studies, the rate of curative resection in EGCs was low with tumors exhibiting undifferentiated microscopic pattern as opposed to differentiated tumors [34, 57–59]. Another peculiar behavior of gastric adenocarcinoma displaying undifferentiated histology (mainly the signet-ring cell carcinoma) is the tendency to extend along the sub-epithelial plane [60]. Hence, a wider safety margin of the surrounding mucosa has to be considered for tumors with poor differentiation.

Regarding macroscopic type of tumors, majority of published data consider the three-tier grouping system of Paris classification: elevated, flat and depressed gross types [61]. But in reality, lesions with mixed endoscopic appearances are commonly seen in clinical practice as shown in Fig. 2. The Japanese classification system [3] provides a better and wider macroscopic description for early gastric neoplasia that includes all pure and mixed variants in their respective category (0-I, 0-II [a,b,c], 0-III and mixed with any one of the types predominating).

In our study, we adopted the Japanese guidelines for macroscopic classification of the EGCs to investigate their association with non-curability after ESD. In concordance with a study done by Ohara et al. [62], our multivariate analysis depicted that EGCs with purely superficial elevated (0-IIa) or depressed (0-IIc) or their mixed gross types with predominant elevated or depressed component (Mixed with 0-IIa or 0-IIc dominant) were significantly associated with NEC resection. In a univariate analysis, Abe et al. [63] recently reported that superficial depressed and/or elevated lesions were found to be indicative of submucosal invasion ≥ 500 μm, even though, their finding was lacking significance in a multivariate regression analysis. Similarly, Yamada et al. found that EGCs with superficial elevated and depressed endoscopic features were greatly linked with submucosal as well as invasion of lympho-vascular structures [64]. The above-mentioned studies are consistent with our results in that superficial elevated or depressed macroscopic types have a predilection to invade the submucosal layer and hence leading to non-curability after endoscopic resection.

It is well known that metastasis to lymph nodes is a very critical condition in determining curability following resection of EGCs. Several previous researchers had also confirmed that EGCs invading deep into the submucosal layer and lympho-vascular structures are independently associated with metastasis to lymph nodes [20, 65–68]. Thus, taking all these ideas together, we can infer that EGCs with superficial elevated or depressed gross type are likely to have metastasis to lymph nodes. And great care is needed while encountering EGCs with purely superficial elevated (0-IIa) or depressed (0-IIc) or their mixed gross types with predominant elevated or depressed component (mixed with 0-IIa or 0-IIc dominant).

In the present study, presence of ulcerative finding was associated with NEC resection in a univariate analysis and it was found to be an independent predictor of submucosal infiltration in patients with EGCs after ESD as shown in multivariate analysis (Table 5). In a multivariate analysis, Ohnita et al. [44] reported that absence of ulceration significantly predicted curative outcomes following ESD (*p* = 0.002). In a similar way, other studies demonstrated the interference of ulcer in curability of endoscopically resected lesions [69, 70]. It has been reported that presence of ulceration, mainly in those with greater than 2 cm size, caused technical difficulty during removal procedure leading to incomplete resection of EGCs [71]. Hence, the procedure of ESD might not be smooth in the setting of ulceration, thus, hindering proper dissection along the submucosal plane resulting in NEC resection.

Our study is not without shortcomings. Firstly, it was a retrospective study and thus a large-scale multi-center prospective cohort data is needed for external validation. Secondly, the number of enrolled subjects was relatively small, especially those cases who had ulceration, positive margins and positive lympho-vascular structures. Lastly, data related to patients’ follow up and lymph node status were unavailable. Consequently, we could not deal with post-ESD tumor recurrences and disease survival conditions.

## Conclusions

We demonstrated that certain tumor-associated factors such as location in upper two third of the stomach, larger tumor size (> 2 cm) and undifferentiated histology are significantly associated with submucosal invasion and NEC outcomes. Therefore, great care is needed while handling EGCs with these clinico-pathologic features. There is very little description in the literature regarding endoscopic macroscopic feature of tumors and their role in curability after ESD by using the JGCA classification. In this study, we discovered that tumors with macroscopic type of purely superficial elevated (0-IIa) or depressed (0-IIc) or mixed gross type with predominant 0-IIa or 0-IIc are highly linked with non-e-curability. Therefore, the findings of our study may shed light on the consideration of certain clinico-pathologic features such as location and macroscopic types in the indication criteria for ESD.

## Data Availability

The datasets used and/or analyzed during the current study are available from the corresponding author on reasonable request.

## Abbreviations

CI: Confidence interval
D: Differentiated
EGC: Early gastric cancer
EMR: Endoscopic mucosal resection
ESD: Endoscopic submucosal dissection
JGCA: Japanese gastric cancer association
M: Mucosal
NEC: Non-e-curative
OR: Odds ratio
SD: Standard deviation
SM: Submucosal
T1a: Intra-mucosal tumor
T1b: Submucosal tumor
U: Ulcer
UD: Undifferentiated
